# *Astragalus membranaceus* Enhances Myotube Hypertrophy through PI3K-Mediated Akt/mTOR Signaling Phosphorylation

**DOI:** 10.3390/nu14081670

**Published:** 2022-04-17

**Authors:** Tzu-Shao Yeh, Tze-Huan Lei, Jen-Fang Liu, Mei-Chich Hsu

**Affiliations:** 1 School of Public Health, Nantong University, Nantong 226019, China; her-email@yahoo.com.tw; 2 College of Physical Education, Hubei Normal University, Huangshi 435002, China; tzehuanlei@gmail.com; 3 Department of Nutrition and Health Sciences, Chang Gung University of Science and Technology, Taoyuan 33303, Taiwan; 4 Department of Sports Medicine, Kaohsiung Medical University, Kaohsiung 80708, Taiwan; 5 Department of Medical Research, Kaohsiung Medical University Hospital, Kaohsiung 80708, Taiwan

**Keywords:** Huangqi, sarcopenia, geriatrics, traditional Chinese medicine, mTOR

## Abstract

*Astragalus membranaceus* (AM) is classified as a high-class traditional herbal medicine, which has strengthened vitality and multifunctional pharmacological activities, but limited empirical evidence is available to support its effects in muscular hypertrophy. It evokes skeletal muscle hypertrophy by increasing anabolic pathway, which is essential to prevent sarcopenia in elderly population. In this study, we examined the effects of AM on skeletal muscle hypertrophy by focusing on the molecular mechanism. We employed an in vitro model to investigate whether AM-treated skeletal muscle, as represented by myotube C2C12 cells, was hypertrophic, and to further investigate the efficacy of AM-activated phosphorylation of PI3K/Akt/mTOR signaling that must occur prior to myotube hypertrophy. The results showed that the myotubes formed larger multinucleated myotubes with increased diameter and thickness (1.16-fold relative to control group, *p* < 0.05). Administration of PI3K and mTOR inhibitors abolished AM-induced muscular hypertrophy. Moreover, AM-induced PI3K-mediated myotube hypertrophy was accompanied by the activation of Akt and mTOR signaling. We concluded that the AM is a nutritional activator to enhance muscular hypertrophy by increasing PI3K/Akt/mTOR signaling phosphorylation. As the AM is effective in myotube hypertrophy, AM and its derivatives may be promising candidates for ergogenic aid to prevent sarcopenia.

## 1. Introduction

Sarcopenia has been defined as an age-related loss of skeletal muscle mass and strength [1]. Clinically, the ideal strategy for an elderly population with loss of muscular mass, force, and function is to combine exercise training, especially resistance exercise, along with nutritional intervention. Some nutrients have been shown to modulate muscular protein metabolism or muscle cell functions, or both [2]. Nutritional intervention such as L-Carnitine, creatine, and leucine [3], branched-chain amino acids, citrulline, arginine, or α-ketoglutarate [4] has been shown to promote anabolic protein metabolism in the human body. However, their effectiveness on muscle mass or strength remains unclear and some of them have even been shown to have adverse effects, such as increased level of hematocrit, worsening of sleep apnea, and increased frequency of cardiovascular accidents [2,4]. Apart from those nutrients mentioned above, some herbal natural active substances are being studied to attempt to prove their efficacy and safety in the elderly population. In recent years, sports nutritionists have been interested in discussing herbal natural actives such as ginsenoside [5], *Epimedium* extract [6], and *Schisandrae chinensis* fructus extract [7] that could promote greater muscular hypertrophy signaling pathway in vitro.

In adult mammals, muscle mass and muscular hypertrophy is a prerequisite for muscular strength [8]. Skeletal muscle hypertrophy is regulated by phosphatidylinositol 3-kinase (PI3K)/Akt/mammalian target of rapamycin (mTOR) pathway [9,10,11]. Activating Akt serine/threonine kinase (also called PKB, protein kinase B) can prevent muscle loss [12]. The phosphorylation of Akt is indispensable for IGF-1 (insulin-like growth factor)-induced hypertrophy [13]. Therefore, the Akt and mTOR signaling is known as a pivotal pathway and it can induce muscle hypertrophy. If a nutritional substance can rapidly activate PI3K/Akt/mTOR signaling-mediated muscular hypertrophy pathway and play an active role in both the number and size of the muscle fibers, it could potentially improve skeletal muscle mass or promote muscular hypertrophy, thereby acting against the age-related sarcopenia.

*Astragalus membranaceus* (AM) is one of the famous Chinese materia medica with the function of increasing human vitality. It has long been customary to use AM as a long-term health supplement to recuperate the weak body [14]. According to modern chemical analysis, AM roots are rich in saponins, amino acids, polysaccharides, flavonoids, and trace elements [15,16]. Amongst many constituents in astragalus root, the most widely discussed is astragalus saponins [17]. Astragalosides have multiple pharmacological activities and are being adopted as a quality-control major component of AM in Chinese Pharmacopoeia [18]. Based on review articles and clinical practices in recent years, astragalosides and other active substance of AM possess a potential for broad application in many clinical diseases [19]. Studies have reported that AM has a cardioprotective effect [20,21,22,23], promotes angiogenesis [24,25], protects endothelial function [26], prevents of ultraviolet A-induced photoaging [27], has hepatoprotective effect [28], improves in insulin resistance [29], and is involved in immunomodulatory activities [30,31,32] function. However, whether AM could potentially enhance muscular function remains equivocal and thus warrants further investigation.

We have previously studied the functions of AM and our results revealed that AM ingestion combined with habitual exercise led to enhanced muscular strength. We also demonstrated a dose-dependence of AM supplementation in mice [33]. Furthermore, some studies revealed that AM can regulate part of the Akt in insulin-resistant skeletal muscle [34] and the mTOR pathway in breast cancer cells [35]. In order to break through the ineffectiveness of traditional use of protein or amino acids as the nutritional supplements in elderly population with sarcopenia, the quality of nutrient supplementation has to be focused on promote muscular hypertrophy. Therefore, we conducted this in vitro study to explore whether the water extract of AM could potentially promote muscular hypertrophy by activating PI3K/Akt/mTOR signaling. We hypothesized that AM could be an ideal nutritional activator to promote muscular hypertrophy by increasing the Akt/mTOR signaling pathway.

## 2. Materials and Methods

### 2.1. Skeletal Muscle Cell Culture

The C2C12 skeletal muscle cell line was purchased from the BCRC (Bioresource Center, Hsinchu, Taiwan). Cells were cultured in growth medium that consisted of DMEM (11965; Invitrogen, CA, USA) supplemented with 10% FBS (10437; Invitrogen, CA, USA) and 1% Pen/Strep/Amph (Biological industries, Kibbutz, Israel) at 37 °C, humidified 5% CO_2_ incubator. When cells reached 90% confluence, the medium was swapped to the differentiating medium, consisting of DMEM supplemented with 2% HS (horse serum) and 1% Pen/Strep/Amph [36,37]. After five days of cell culture, the C2C12 cell was matured to striated myotubes and assigned to three groups for subsequent experiments [38]. For the control group, the myotubes were maintained in DMEM + 2% HS (NON); for the positive control group, the myotubes were cultured in DMEM + 2% HS supplemented with IGF-1 (concentrations as 10 ng/mL; IGF-1); and for the experimental group, the myotubes were cultured in DMEM + 2% HS supplemented with AM (concentrations as 10 ng/mL; AM).

### 2.2. Astragalus membranaceus (AM) and Chemical Reagents

Reagents were obtained from commercial sources: AM (10 mg/mL) were from International Total Solution Taiwan (Taipei, Taiwan); wortmannin (inhibitor of PI3K, W1628), rapamycin (inhibitor of mTOR, R0395), and IGF-1 (I8779) were from Sigma-Aldrich. Stock solution of AM and the chemicals were dissolved separately in phosphate-buffered saline (PBS, pH 7.40) and aliquoted, then kept at −20 °C freezer. Working solutions were prepared by diluting the stock solution with DMEM medium to the final concentrations as 100 nM wortmannin, 10 ng/mL rapamycin, or 10 ng/mL IGF-1.

### 2.3. Cytotoxicity of Astragalus membranaceus (AM)

The myotubes were exposed to AM (1–10^6^ ng/mL) for 24, 48, or 72 h and cytotoxicity was determined using XTT assay (Biological Industries, Beth Haemek, Israel) in a flat 96-well plate. After 2 h of incubation, the myotube viability was checked by measuring the absorbance at 490 nm using an ELISA microplate reader (550 Bio-Rad, Hertfordshire, UK). Each experiment was performed in quadruplicate. The CVs for repetitive assay (*n* = 4) were less than 6% for control and AM (1–10^6^ ng/mL).

### 2.4. Myotube Diameter

To confirm the effect of AM-induced muscular hypertrophy on myotubes, the myotubes were treated with fresh medium (NON), IGF-1 (10 ng/mL, as positive control; IGF-1), or AM (10 ng/mL; AM) for 72 h and hypertrophic variability in the myotubes were determined under a light microscope (×40 magnification) after treatment. Furthermore, to determine the relevance of AM-induced myotube hypertrophy on hypertrophic signaling, the serum-starved C2C12 myotubes were pre-treated for 30 min with the indicated inhibitors (wortmannin or rapamycin) prior to AM stimulation. Subsequently, cells were washed with cold PBS to remove the inhibitors, then incubated with the aforesaid medium (NON, IGF-1, and AM) for 72 h at 37 °C. Each group was performed in triplicate, and randomly captured by three microscope images from each experimental condition. At least 90 myotubes in each group were analyzed on average from 3 short-axis measurements taken along their length of each myotube by using image analysis software (Image-Pro; Bethesda, MD, USA) then averaged.

### 2.5. Western Blotting

Based on the results of the cytotoxicity, the myotubes were cultured in medium with AM (10 ng/mL) to investigate whether Akt and mTOR phosphorylation was promoted by AM. This trial used a time course experiment to identify the time period for the peaks of phospho-specific of Akt and mTOR. According to the most significant time point from time-course experiments, the myotubes were treated with AM, IGF-1, and fresh medium. Three groups were compared with 1 μM wortmannin (for 30 min to inhibit the PI3K target of signaling pathway). Following the treatment, myotubes were washed two times with cold PBS and scraped down into an Eppendorf tube with protease inhibitors and phosphatase inhibitors (Sigma-Aldrich) for Western blot analysis of the protein level of phospho-Akt on Ser^473^ (p-Akt) and phospho-mTOR on Ser^2448^ (p-mTOR) (Cell Signaling Technology, Beverly, MA, USA).

Quantitation of total protein in a sample was identified by using the BCA (bicinchoninic acid) protein assay reagent (Bio-Rad Lab., Hercules, CA, USA). Aliquots of denatured protein (50 μg) were loaded on SDS-PAGE and transferred to a nitrocellulose membrane. Membranes were incubated overnight with primary antibody with gentle shaking. The primary antibodies, total Akt (t-Akt; 9272; CST), total mTOR (t-mTOR; 2972; CST), p-Akt (9271; CST), p-mTOR (2971; CST), and β-actin (A5441; Sigma-Aldrich, St. Louis, MO, USA), were diluted to a ratio of 1:500 in tris-buffered saline/Tween 20 with 5% NFDM (except β-actin, which was 1:10,000 dilution). Secondary antibodies, anti-mouse IgG (A9044; Sigma-Aldrich) or rabbit IgG (A0545; Sigma-Aldrich), were diluted to a ratio of 1:16,000 in tris-buffered saline/Tween 20 with 5% NFDM, and subsequently used for detection. The protein bands were visualized using ECL (Pierce, Rockford, IL, USA) and quantified using image analysis software (Image-Pro).

### 2.6. Astragalus Analysis of Astragalus membranaceus

Quantitative analysis of AM was carried out with ChromaDex (Irvine, CA, USA). The samples of AM were obtained from the biotechnology company (International Total Solution Taiwan, Inc., Taipei, Taiwan). Astragaloside standards (astragaloside I, II, III, and IV) were purchased from ChromaDex Inc. (Irvine). Samples and standards were dissolved in methanol and filtered through a 0.45 μm filter, and the filtrate was collected in HPLC vial for subsequent analysis. The mixed standard was further diluted as this was necessary to establish the standard calibration curve. The HPLC separation was performed with a polyethylene glycol column by gradient elution. The gradient elution consisted of an initial composition of 5% acetonitrile (HPLC grade, Fairfield, OH, USA) and 95% water (Milli-Q; Milford, MA, USA). The results of the astragaloside compound in AM are illustrated in Table 1.

### 2.7. Statistical Analyses

**Figure 1:**
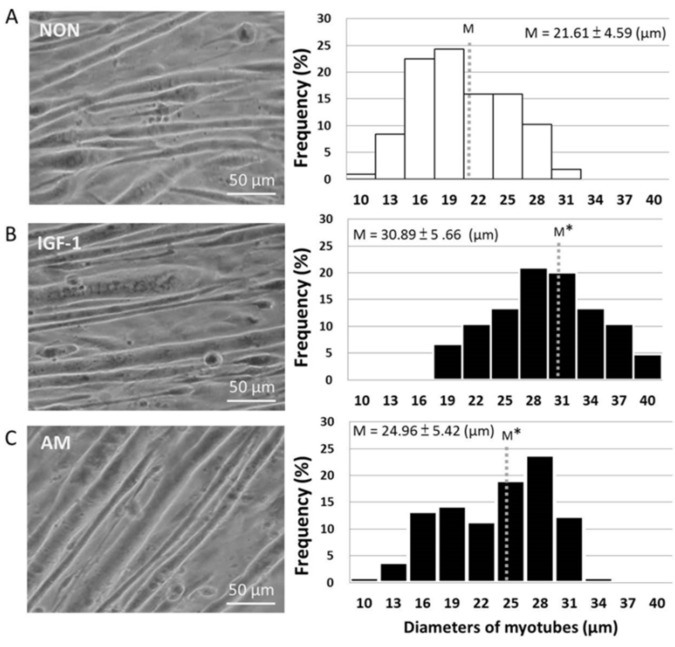
*Astragalus membranaceus* enhances skeletal muscle hypertrophy. Micrographs of representative myotube diameters shown on left panel. Average myotube diameter was quantified along with the length of a selected myotube diameter by image analysis software tools (*n* > 105 per group). (**A**) NON, non-AM supplements; (**B**) IGF-1, insulin-like growth factor-1 treated, 10 ng/mL, as positive control; (**C**) AM, Astragalus membranaceus treated, 10 ng/mL. The mean of myotube diameters (M) was greatly enlarged in IGF-1 and AM group. Student’s *t*-test was employed to examine the average values among groups. Statistical significance: * Indicates significant difference (*p* < 0.05) when compared to the NON.

**Figure 2:**
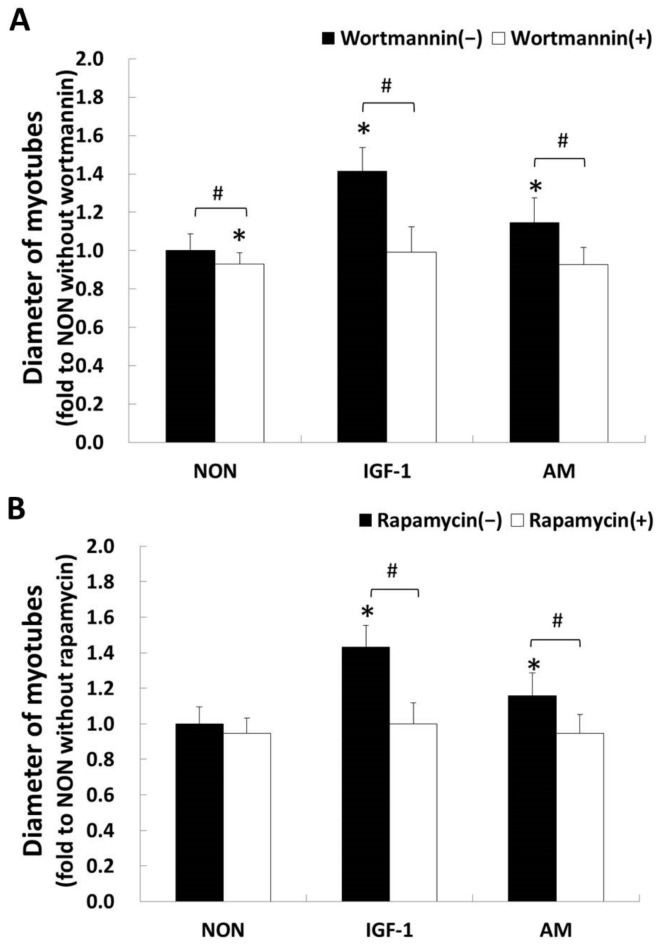
Contribution of linear PI3K/Akt/mTOR signaling in AM-induced myotube hypertrophy. To investigate the potential involvement of PI3K/Akt/mTOR signaling, myotubes were pretreated with (open square) or without (closed square) inhibitors (wortmannin and rapamycin) in DMEM for 30 min, and then swapped to the medium with NON, IGF-1, or AM, respectively, for 72 h of incubation (error bars: SD). Panel (**A**) shows the effects of 100 nM wortmannin in three groups; (**B**) shows the effects of 10 ng/mL of rapamycin in three groups. The vertical axes of the graphs indicate the myotube diameter being normalized to the average diameter of NON without an inhibitor group. NON, non-AM supplements; IGF-1, treated with 10 ng/mL IGF-1; AM, treated with 10 ng/mL AM supplements. Data were examined by two-way factorial repeated ANOVA with Scheffe multiple comparison in (**A**,**B**). * Significant difference (*p* < 0.05) when compared to the NON group without inhibitors: wortmannin (**A**), rapamycin (**B**). # Significant inhibitory effect in the same group (*p* < 0.05).

**Figure 3:**
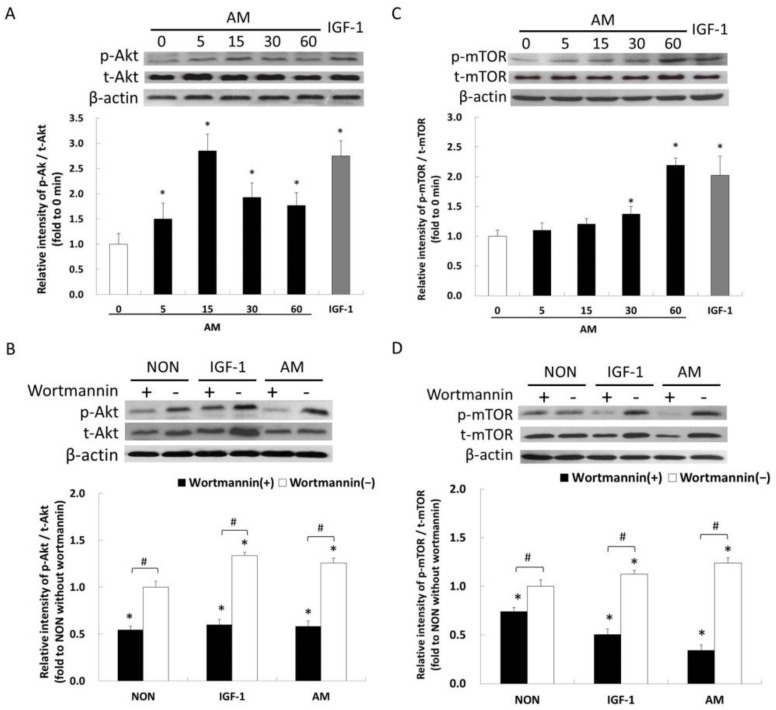
Phospho-Akt Ser^473^/mTOR Ser^2448^ induced by AM. (**A**) Time-course analysis of phospho-Akt/total-Akt protein levels in the myotubes stimulated 10 ng/mL of AM. (**B**) The levels of Akt and its phosphorylated form at 15 min of AM-treated in specific PI3K inhibitor-treated myotube. β-actin was probed to monitor equal loading. (**C**) Time-course analysis of phospho-mTOR/total-mTOR protein levels in the myotubes stimulated with 10 ng/mL of AM. (**D**) The levels of mTOR and its phosphorylated form at 60 min in wortmannin-treated myotubes. Phospho-mTOR and total-mTOR were normalized by individual β-actin. Results represent mean ± SD of three independent experiments. Data were examined with one-way analysis of variance with time factors in (**A**,**C**). Data were examined with two factorial repeated analysis of variance (groups and inhibitor treatment) (**B**,**D**). * *p* < 0.05 vs. treat-AM at 0 min in (**A**,**C**) (Scheffe test). * *p* < 0.05 vs. NON without inhibitor wortmannin in (**B**,**D**) (Scheffe test). # Indicates significantly different (*p* < 0.05) within group for the comparison of the same group (Scheffe test).

Statistical analysis was performed using the commercially available software (SPSS version 14; Chicago, IL, USA), with the significance level set at *p* < 0.05. All values were presented as mean ± SD. Comparisons of myotube diameters between groups (Figure 1) were assessed by Student’s *t* test. Groups and inhibitor treatment data (Figure 2 and Figure 3B,D) were examined by using two-way factorial ANOVA combined with Scheffe’s post hoc analysis. The difference in relative protein intensity data between various time- point treatments (Figure 3A,C) were analyzed by using one-way repeated ANOVA with Scheffe multiple comparison. The data on the cytotoxicity of myotubes were analyzed to detect dose effects of AM concentration (Table 2) by using one-way ANOVA.

## 3. Results

### 3.1. Cytotoxicity of Astragalus membranaceus on Myotubes

To examine different concentrations and time course effects of AM on the cytotoxicity of myotubes, percentage of viable myotubes were determined by XTT assay. The viability of myotube in the control group was represented as 100%. As shown in Table 2, compared with the untreated control, the percentage of viable myotubes kept within a certain range of concentration, especially at the concentrations of 1 and 10^4^ ng/mL. AM at a concentration of 10^5^ ng/mL caused a cytotoxic response, and approximately 12% of the myotubes lost their viability within 48 h of AM treatment. Increasing the AM concentration to 10^6^ ng/mL caused a more toxic response, and over 18% of the myotubes lost their viability after incubating for the same time. Similar results were obtained in the cytotoxicity of myotubes induced by 72 h of AM treatment. The results indicated that AM was non-toxic to myotubes at concentrations from 1 to 10^4^ ng/mL for 24, 48, and 72 h.

### 3.2. Promotion of Myotube Hypertrophy by Astragalus membranaceus

To determine whether AM enhanced myotube hypertrophy, we examined the effect of AM on myotube thickness. Our results revealed that AM directly resulted in myotube hypertrophy. This was evidenced by highly thickened myotubes in the IGF-1 (positive control, 30.89 ± 5.66μm) and AM-treated groups (24.96 ± 5.42μm) after 72 h of incubation, compared with the NON control group (21.61 ± 4.59μm). The myotube diameters of the three groups (NON, IGF-1, and AM) presented normal distributions. The result indicated that the average diameters of myotube in the IGF-1 and AM groups were 1.43- and 1.16-fold higher when compared to the NON group (Figure 1).

### 3.3. Astragalus membranaceus and the PI3K/Akt/mTOR Pathway

We employed pharmacological inhibitors of PI3K or mTOR that blocked the PI3K/Akt/mTOR signaling to determine the participation of this pathway in AM-induced myotube hypertrophy. IGF-1 was used as a positive control to the specific activated myotube hypertrophy signaling pathway in this study. Our data revealed that the PI3K/Akt/mTOR signaling has a pivotal role in AM-induced myotubes hypertrophy. This was given the fact that wortmannin, a PI3K inhibitor, reduced the AM- and IGF-1-induced myotube hypertrophy by 20% and 30%, respectively (all *p* < 0.05, Figure 2A). The rapamycin, an inhibitor specific for mTOR, achieved similar results to that of the wortmannin group (Figure 2B).

### 3.4. AM-Induced Phosphorylation of Akt on Ser^473^

Based on the above evidence that the PI3K/AKT/mTOR signaling is involved in AM-induced myotube hypertrophy, we examined whether AM promotes the phosphorylation of Akt. The first part of this trial offers an overview of phospho-Akt protein expression to AM treatment using time-course analysis, which revealed that 5, 15, and 30 min of AM treatment greatly increased the phosphorylated Akt levels (*p* < 0.05, Figure 3A). On the basis of the phospho-Akt expression evident at 15 min of AM treatment, we examined whether Akt phosphorylation is responsive to AM treatment through the PI3K pathway by treating myotubes with a specific PI3K inhibitor, wortmannin, at concentrations of 100 nM. Results revealed that phospho-Akt induced by 15 min of AM stimulation greatly reduced by wortmannin when compared to the NON level (*p* < 0.05, Figure 3B). Thus, the AM-induced phosphorylation of Akt involves a PI3K-dependent mechanism, as presented in the condition of IGF-1 stimulation.

### 3.5. AM-Induced Phosphorylation of mTOR on Ser^2448^

This process is similar to the time-process analysis mentioned earlier. The amounts of phosphorylated mTOR on Ser^2448^ were significantly elevated at 30 and 60 min of AM treatment (*p* < 0.05, Figure 3C). In subsequent experiments, inhibition of PI3K significantly reduced the increase in AM-induced mTOR phosphorylation compared with that of the NON group (*p* < 0.05, Figure 3D). The AM-induced phosphorylation of mTOR involves a PI3K-dependent mechanism. The above results reveal that the PI3K/Akt Ser^473^/mTOR Ser^2448^ signaling may partially regulate AM-induced myotube hypertrophy. However, this does not rule out the possibility that other biological activities regulated by the AM might have also participated in myotube hypertrophy.

## 4. Discussion

To date, the influence of AM on skeletal muscle fibers hypertrophy and enhancing skeletal muscle mass remains equivocal. In this experiment, we examined the effect of AM on myotubes proliferation and its effect on muscular hypertrophy. The present study has three novel findings: (1) AM supplementation greatly enhanced myotube thickness, (2) AM-induced myotube hypertrophy is attenuated when the PI3K/Akt/mTOR pathway is blocked, and (3) AM-induced Akt and mTOR phosphorylation is through the PI3K signaling.

We investigated PI3K/Akt/mTOR signaling to elucidate the molecular mechanisms of AM-induced hypertrophy. Activating this pathway can enhance muscular hypertrophy and maintain muscle structure and force production [39], while IGF-1 is essential for regulating the Akt/mTOR signaling in skeletal muscle hypertrophy [40,41]. Previous studies have not investigated the molecular mechanism of AM-induced skeletal muscle hypertrophy. This study employed Western blotting with specificity antibodies activated for total Akt and mTOR indicated that AM-activated the PI3K/Akt/mTOR signaling pathway in myotubes. This result elucidates the AM hypertrophic effects on myotubes.

This study used two pharmacological inhibitors, wortmannin and rapamycin, to distinguish whether myotubes thickness/hypertrophy is linked with PI3K-regulated Akt/mTOR signaling pathway, or through an alternative pathway. The present study showed that a specific PI3K inhibitor, wortmannin, blocked the effects of AM on myotubes thickness/hypertrophy (Figure 2A). For experiments with the inhibitor specific for mTOR, data behaved similarly to the PI3K inhibitor (Figure 2B). These results indicated that the PI3K/Akt/mTOR signaling played a pivotal role in AM-induced myotube hypertrophy. Dennis et al. [42] revealed that mTOR is a direct target of Akt kinase and determined Ser^2448^ as the Akt specific down-regulation site in mTOR. This result coincided with our study in that we observed that 30 min of AM stimulation enhanced the mTOR phosphorylation level at Ser^2448^ following Akt phosphorylation (Figure 3).

In addition to the regulation of mTOR through the PI3K/Akt signaling, previous studies revealed that the Ser^2448^ phosphorylation also reflects the feedback signal to mTOR from its downstream target, p70S6 kinase (p70^S6K^) [43,44,45,46,47]. The negative regulation of muscular hypertrophy via the p70S6 signaling was a possible reason that we observed an increase in phospho-mTOR at the Ser^2448^ site between 30 and 60 min (Figure 3C). Certainly, we cannot rule out the possibility that AM directly activates p70s6k and feedback signals to mTOR. This requires measuring AM-induced mTOR downstream p70s6k target to answer this open question in future experiments. Although we did not fully elucidate whether AM supplementation could activate mTOR independent of Akt activation, our results at least suggest that with regard to the cell types examined in this study, mTOR at Ser^2448^ phosphorylation exhibited both direct and indirect reactions by AM stimulation and markers of Akt activation.

The Akt serine-threonine kinases (also called protein kinase B) are key regulatory proteins involved in adult skeletal muscle induces hypertrophy. Many previous studies reported that the activation period of phospho-Akt on Ser^473^ is relatively transient after a nutritional stimulation for activation [5,46,47,48,49,50,51,52]. In our study, the phospho-Akt on Ser^473^ was found as early as after 5 min of AM treatment and increased within 15 min to its maximum. The change trend of phosphorylation Akt after nutritional stimulus on myotube is similar to the findings from previous studies [46,48].

One previous publication addressing AM signaling in skeletal muscle cell lines also revealed mTOR as a target for inhibiting apoptosis of myoblasts and its potential contribution in protecting skeletal muscle atrophy [51]. Lu et al., observed in a muscle cell line (C2C12) that mTOR phosphorylation increased after 1 h of Astragalus polysaccharide stimulation, a time lapse that was similar to our results in mTOR on Ser^2448^ phosphorylation by using AM stimulation. Two recent publications on natural triterpenoids showed that C2C12 cells treated with ginsenosides [5] or black ginseng [52] both triggered the phosphorylation of Akt and enhanced the expression of MyoD and myogenin inducing myogenic conversion of fibroblasts to recover the capacity of muscular regeneration. These results partially supported our results that AM with natural triterpenoids could induce myotube hypertrophy via the Akt/mTOR signaling.

Muscle growth in many conditions shares a common mechanism, the Akt/mTOR pathway is upregulated during muscle hypertrophy and downregulated during muscle atrophy [12]. Although the findings obtained from our results with increasing phospho-mTOR protein level in myotubes may explain the Akt phosphorylation state, the mechanism on the sustained increase of Akt levels remains unclear. Available evidence supports a role for risen phosphorylation of Akt not only in muscle hypertrophy, but also in catabolic or atrophic conditions [38]. A limitation of our experiments is that we have not yet clarified whether AM nutritional intervention is associated with downstream regulatory sites of Akt/mTOR in skeletal muscle atrophy, such as the MuRF1 and MAFbx/Atrogin-1. Meanwhile, an expanding body of evidence suggests that sarcopenia is mechanistically different from acute atrophies induced by disuse, disease, and denervation [53,54]. As the link between the disease- and disuse-induced muscle atrophies and aged sarcopenic muscle is conflicting, more evidence is needed to clarify the mechanism differences between those two. We would like to concentrate the effect of AM on Akt/mTOR pathway first before resolving the issue of sarcopenia, since the level of Akt/mTOR phosphorylation is considered to be a good predictor of increased muscle hypertrophy [5,9]. Activated Akt/mTOR is not only capable of increasing diameter of the normal skeletal muscle fibers, but also capable of preserving myofiber size in aged sarcopenic muscles that undergo atrophy [12]. Furthermore, we used two specific blockers in normal myotube models tested, without causing atrophy in the control muscles. By identifying a crucial signaling pathway responsible for regulating skeletal muscle hypertrophy, our results certainly contribute to the development of nutritional therapies for upregulating myotube hypertrophy.

## 5. Conclusions

In this study, we demonstrated that AM rapidly activates the Akt/mTOR pathway through PI3K and subsequently induces skeletal muscle hypertrophy in cultured myotubes. As far as we know, this is the first evidence to demonstrate that the PI3K/Akt/mTOR pathway in myotube hypertrophy induced by AM treatment is similar to the outcome of IGF-1-induced hypertrophy. Our findings provide empirical evidence to support the current prevailing view that AM treatment can strengthen vitality. As the AM is effective in myotube hypertrophy, AM and its derivatives may be promising candidates for the ergogenic aid to prevent sarcopenia.

## Figures and Tables

**Table 1:** The astragaloside compound in *Astragalus membranaceus* for this study.

Identification	*Astragalus membranaceus* Sample (mg/g)
Astragaloside I	0.212
Astragaloside II	0.431
Astragaloside III	0.076
Astragaloside IV	0.507
Total Astragalosides	1.226

Astragaloside I, Astragaloside II, and Astragaloside IV were the main compounds in *Astragalus membranaceus* of this study. BRL, the compound detected below reporting limit. Astragalosides analysis by high performance liquid chromatography with charged aerosol detector (HPLC/Corona-CAD) chromatogram. The chromatographic separation was performed a Phenomenex Kinetix C18 column (150 × 4.6 mm, 2.6 μm, 100 Å), and the mobile phase with the gradient elution at a flow rate of 0.9 mL/min at 40 °C. Injection volume was 5 μL.

**Table 2:** Effect of *Astragalus membranaceus* on viability of myotubes (%).

		Concentration of Treated *Astragalus membranaceus* (ng/mL)						
	Con	1	10	10^2^	10^3^	10^4^	10^5^	10^6^
24 h	100.00 ± 4.69	98.44 ± 5.41	97.25 ± 4.45	97.23 ± 4.16	96.36 ± 5.03	93.18 ± 3.02	89.56 ± 1.33	87.94 ± 4.11
48 h	100.00 ± 4.74	100.59 ± 4.88	101.19 ± 4.23	100.08 ± 3.78	97.38 ± 3.31	92.99 ± 3.01	88.15 ± 2.24 *	81.91 ± 4.19 *
72 h	100.00 ± 3.75	99.25 ± 3.49	101.31 ± 5.21	99.34 ± 4.86	99.10 ± 3.92	92.56 ± 3.59	88.11 ± 5.26 *	82.64 ± 4.53 *

Different concentrations and time course effects of AM on myotubes cytotoxicity were determined by XTT assay in quadruplicate. Values were expressed as mean ± SD. Data were examined by one-way repeated ANOVA for different doses. * *p* < 0.05 compared with data from a control group at the same time of experimental group.
